# Characteristics of Disease Relapses and Their Relationships with Weather Conditions in Patients with Multiple Sclerosis

**DOI:** 10.3390/jcm14227960

**Published:** 2025-11-10

**Authors:** Izabela Sempik, Anna Pokryszko-Dragan, Małgorzata Wieczorek, Marek Błaś, Edyta Dziadkowiak

**Affiliations:** 1 Department of Neurology, Regional Hospital in Legnica, Iwaszkiewicza 5, 59-220 Legnica, Poland; sempikiza@gmail.com; 2 Clinical Department of Neurology, University Centre of Neurology and Neurosurgery, Wroclaw Medical University, Borowska 213, 50-556 Wroclaw, Poland; anna.pokryszko-dragan@umw.edu.pl; 3 Institute of Geography and Regional Development, University of Wroclaw, Uniwersytecki Square 1, 50-137 Wroclaw, Poland; malgorzata.wieczorek@uwr.edu.pl (M.W.); marek.blas@uwr.edu.pl (M.B.)

**Keywords:** multiple sclerosis, relapsing-remitting, meteorological parameters, weather pattern

## Abstract

**Background**: The aim of the study was to analyze the clinical characteristics and circumstances of relapses in the patients with relapsing-remitting multiple sclerosis (MS). **Objectives**: The eighty patients with clinically definite MS and relapsing-remitting course were enrolled in the retrospective study. **Methods**: The calendar of documented recurrences was analyzed, looking for any patterns across years, warm and cold periods, and seasons and months. **Results**: In the years 2015–2020 the majority of relapses occurred in March, June–July, and October; with regard to seasons, the relapse rate peaked during spring and summer. In 2021–2023 there was significant increase in relapses in May and in February. In these years, most cases occurred in spring, and the least in autumn. The most significant coincidences were found for sensory symptoms in January, optic neuritis in March, motor deficit with pyramidal signs in May and June, cerebellar symptoms in March and July, and spinal cord involvement signs in August. **Conclusions**: Observation of seasonal occurrence of relapses revealed periods with high temperature, low humidity, and variable atmospheric pressure as potential contributors. Better recognition of these issues within future investigations could be considered in the complex approach to the management of MS outcomes.

## 1. Introduction

Multiple sclerosis (MS) is a chronic autoimmune inflammatory disease of the central nervous system (CNS). The most prevalent MS phenotype (seen in 85–90% of the patients) is the relapsing-remitting one. It is characterized by exacerbations of inflammatory activity in the CNS, clinically manifested as relapses, separated with periods of clinical stability (remissions). In almost half of cases, relapses result in residual deficits, contributing to relapse-associated worsening (RAW), which leads to cumulative disability and clinical deterioration in the course of disease [1,2].

According to the standard definition—MS relapse is indicated by episodic appearances of neurological deficits (new symptoms and signs or aggravation of previous ones), lasting at least 24 h, and occurring after a period of at least 30 days of stability or improvement. With the use of the Expanded Disability Status Scale (EDSS) as a measure of neurological deficit, relapse is usually defined by increase in EDSS score by at least 0.5 or 1.0 points (with baseline EDSS > 5.5 or <5.5, respectively) [3]. The most common symptoms of relapse include sensory impairment, visual disturbances, motor deficit, and balance problems. In approximately 74% of cases, they are monofocal and correspond with the involvement of the optic nerve, brainstem, cerebellum, or spinal cord. In some cases, the symptoms of relapse are multifocal, which is assigned as dissemination in space and has diagnostic and prognostic relevance [1,2,4]. Recently, attention has been paid to specific phenotypes of relapse, associated with acute cognitive deterioration or increase in fatigue; yet their appropriate recognition requires application of specific tools (e.g., standardized neuropsychological tests) and long-term observation of these aspects of MS-related CNS dysfunction [5,6,7,8,9]. Symptoms and signs of relapse are completely or partially resolved, usually within several days (spontaneously or after treatment). Overall, relapses may differ in type of symptoms, severity, speed of onset, duration, and recovery not only in the MS population, but also in individual patients throughout the disease [6,10].

Identification of relapses may become difficult if previous symptoms deteriorate after incomplete remission or if they are superimposed on the gradual progression of disability (conversion into secondary progressive course) [11,12]. Relapses should also be distinguished from pseudorelapses or fluctuations of symptoms which may occur, e.g., during overheating (Uthoff’s phenomenon) or infection, usually only temporarily and concomitantly with predisposing conditions. However, several factors have been identified which may precede and trigger the relapse (including infections, vaccines, surgery, mental stress, extreme physical effort, or hormonal imbalance—e.g., delivery and post-partum period). Although relapses are defined in clinical terms, integrating clinical assessment with the evaluation of new and contrast-enhanced lesions in magnetic resonance imaging (MRI) may contribute to better recognition of disease activity [12,13,14,15].

The standard treatment for MS relapses is high doses of methylprednisolone in IV infusions; plasmapheresis may be recommended in selected cases of corticosteroid insufficiency [2]. Regarding disease-modifying therapies (DMTs), their main goals include reduction in relapse rate and severity, as well as prevention of relapse-related disability worsening. It seems that avoiding or modifying potential triggering factors would further reduce a relapse risk, in addition to effects of DMTs. Defining such triggering factors, associated with environmental or lifestyle issues, might allow us to address them in overall prevention, consistent with complex therapeutic approaches currently recommended to MS patients [1,2,5,7,16].

The aim of the study was to analyze the clinical characteristics and circumstances of relapses in the patients with relapsing-remitting MS, particularly considering their seasonal occurrence and association with weather conditions, to identify the spectrum of potential triggering factors for relapses.

## 2. Materials

The study was conducted in the Department of Neurology, Specialistic Hospital in Legnica. Participants were recruited from the patients with MS who attended regular outpatient follow-up in the department due to disease-modifying treatment (DMT). 

Inclusion criteria comprised the diagnosis of relapsing-remitting MS (according to McDonald’s criteria—revision 2017) [3], regular follow-up for at least 2 years, with at least one relapse documented during this period, and available complete medical records reporting the course of disease and treatment.

Exclusion criteria included patients younger than 18 years, progressive forms of MS, coexisting uncompensated chronic conditions (e.g., diabetes, hypertension, and renal or liver failure), or other CNS co-morbidities.

Finally, 80 patients were recruited. The ultimate study group included 59 females (73%) and 21 males (27%), with the mean age of 43 years.

## 3. Methods

The design of the study was retrospective, based on analysis of the patients’ medical records.

### 3.1. Demographic and Clinical Characteristics

Demographics (age and gender) and clinical MS-related data (duration and course of the disease, current neurological status, degree of disability in Expanded Disability Status Scale, and type of DMT) were established on the basis of medical records. The mean disease duration in the study group was 10 years, and the median EDSS was 2.29 for women and 2.5 for men. A total of 54 women (91%) and 20 men (95%) were receiving currently available disease-modifying therapies (DMTs). Moderate-efficacy (ME) treatments included interferon beta (19 patients), glatiramer acetate (6 patients), dimethyl fumarate (21 patients), and teriflunomide (9 patients). High-efficacy (HE) treatments included fingolimod (4 patients), natalizumab (2 patients), ofatumumab (6 patients), ponesimod (1 patients), and ocrelizumab (6). The mean duration of DMT use was 6.2 ± 4.4 years.

The relapses within the documented follow-up were identified in participants. All the events qualified as relapses were confirmed by a neurologist during inpatient or outpatient consultation, including physical examination and assessment on the EDSS. In some cases, MRI was additionally performed during a relapse, but due to limited availability of these findings, they were not included in analysis. The following information concerning a relapse was determined: reported date of the relapse onset, clinical manifestation (symptoms and signs), and the presence of preceding/triggering factors (infection, including COVID-19, other acute conditions, surgical procedures, vaccination, non-health-related stressful event, and pregnancy/delivery)—reported by the patients or documented with additional medical records. In the next stage, an analysis is carried out to find the relationships between specific symptoms of relapse and age, gender, duration of disease, and DMTs.

### 3.2. Climate Data

At the beginning, we analyzed a calendar of documented relapses, looking for any regularities throughout years, seasons, and months. The period of the SARS-CoV2 pandemic was distinguished due to a significant increase in prevalence and exposure to infections in general and in the MS population. Therefore, we adopted a division of the analyzed timeframe in two periods: 2015–2020 and 2021–2023. Each relapse was assigned to one of the 12 calendar months, 4 seasons, and 2 periods (warm and cold), according to the time of its occurrence, based on medical records. On this basis, an analysis was carried out, taking into account the relationship between specific symptoms of relapse and a month or a season of its occurrence.

Moreover, for each season with a relapse occurrence, average values were estimated for the set of basic meteorological parameters (e.g., maximum and minimum air temperature with daily amplitude, relative humidity, atmospheric pressure, insolation, and wind speed). In the next step, the average amplitudes of changes in meteorological parameters that occurred in 2-, 3-, 4-, 5-, and 6-day cycles were calculated. Finally, 52 monthly average values were calculated for each month with a relapse and were compared with their equivalents for the days directly preceding the onset of relapse symptoms. The differences between monthly averages and daily quantities have been standardized by referring them to the multiple of the standard deviation (SD). The aim of this procedure was to check whether the days associated with the occurrence of MS relapses were similar to the average weather conditions in the given month or if they stood out from this background. On this basis, a specific weather pattern corresponding with a relapse occurrence was determined.

In addition, a correspondence analysis was carried out taking into account the time of occurrence and symptoms of relapse simultaneously, to investigate multidimensional relationships between these data.

### 3.3. The Statistical Analysis

Descriptive statistics were performed as the first step of the analysis. The Shapiro–Wilk test was used to test the distribution of the data. Student’s t and the Mann–Whitney U test allowed us to verify differences between women and men in terms of age and duration of disease. A Z-score ratio test was performed when the counts of individual fractions >10 or the Chi-square test with Yates correction, respectively, when the count criterion was not met. Correspondence analysis was used to explore the association between age group and symptom type. The Ward clustering method with Euclidean metrics allowed us to indicate detailed relationships between the co-occurrence of these two features (age and symptom). The visualization of the relationships was presented using a dendrogram. The same method was used to analyze the occurrence of given symptoms depending on the month. 

The analyses and dendrograms were performed using Statistica version 13.3. The graphs were drawn in MS Office 356.

## 4. Results

### 4.1. Relapse Characteristics

Analyzing the medical records of the patients, we determined 80 relapses placed between 2015 and 2023, for which the investigated data (symptoms, potential triggering factors, time of occurrence, and treatment) were available.

The most common symptoms of relapse were those of motor deficit with pyramidal signs—38; optic neuritis—14; and cerebellar dysfunction—10, followed by spinal cord involvement signs—8; sensory impairment—7; brainstem symptoms—2; and 8th nerve palsy—1, respectively. In 15 cases, relapse was the first clinical manifestation of MS, assigned as clinically isolated syndrome (CIS); in all these cases CIS was monofocal and included motor deficits with pyramidal signs, spinal cord involvement signs, or optic neuritis.

In the analysis of the relationships between symptoms and gender, the most significant finding was that sensory symptoms were recorded only in women. Motor deficit with pyramidal signs was observed more often in men (57%) than in women (44%). A percentage of 20% of women experienced optic neuritis, while almost 20% of men presented with spinal cord involvement signs (Table 1).

A correspondence analysis was performed of the relationships between the type of relapse symptoms and the age of patients. In the group of the youngest patients (<30 years old) sensory impairment and optic neuritis predominated. Those between 30 and 40 years of age presented mostly with motor deficits with pyramidal signs and optic neuritis. The most numerous group of patients, older than 40 years, experienced mainly motor deficits with pyramidal signs (Table 2).

### 4.2. Seasonal Occurrence of Relapses

Due to specificity of pandemic SARS-CoV2, we analyzed the occurrence of relapses in two periods: 2015–2020 and 2021–2023. Indeed, there was a marked increase (ca. threefold) in the number of relapses after 2020, in comparison with the previous period. Until 2020, the rate of relapses was approximately five per year, while in 2021, 16 events were noted, and later the rate was ca. 15 per year.

**Figure 1:**
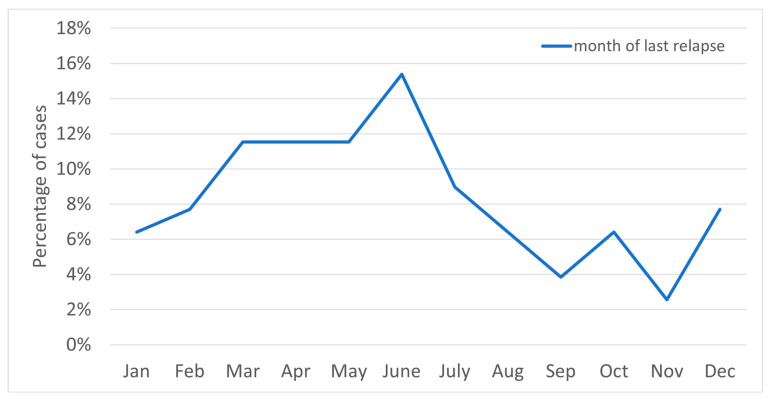
The chart above shows the relapse frequency by month.

**Figure 2:**
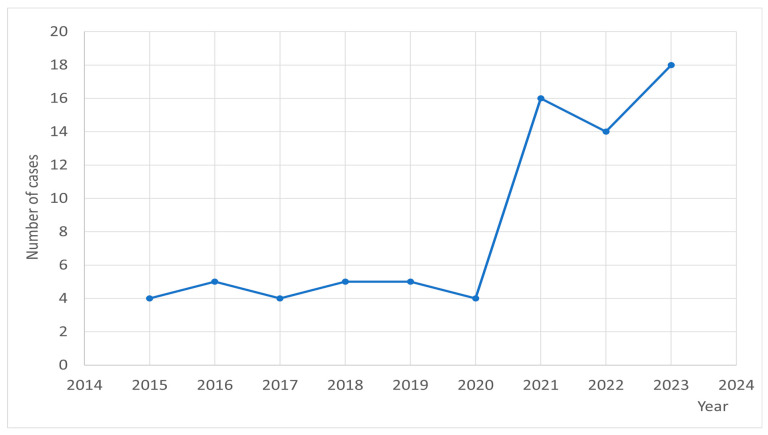
Relapses according to date of occurrence.

Figure 1 shows the relapse frequency by month; Figure 2 shows relapses according to date of occurrence.

**Figure 3:**
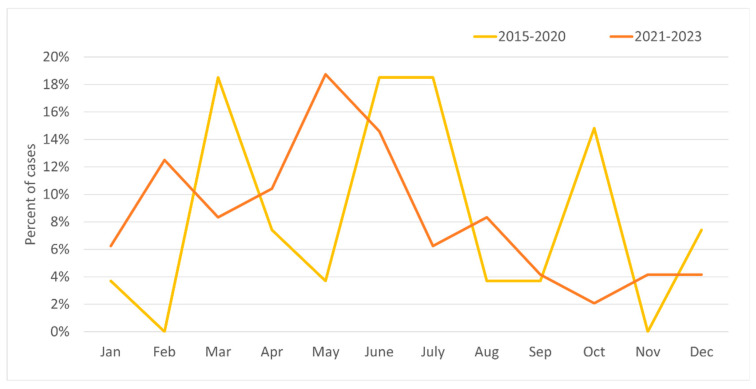
Percentage of incidence of MS relapses by month for the study group in two periods: 2015–2020 and 2021–2023.

**Figure 4:**
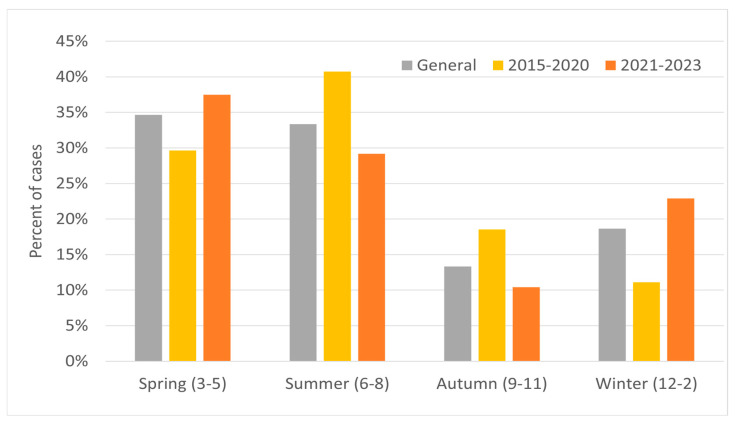
Percentage of MS relapses by season for the study group in two periods: 2015–2020 and 2021–2023.

In the years 2015–2020, the majority of relapses occurred in March, June–July (18–19% for each), and October (15%); with regard to seasons, the relapse rate peaked during spring and summer. In 2021–2023 there was significant increase in relapses in May (19%) and in February (12%), while for the other months the value was 2–7% (Figure 3). In these years, most cases occurred in spring, and the least in autumn (Figure 4). Taking into account the division of months into warm and cold seasons, there was a clear dominance of relapse occurrence in warm months in both analyzed periods (more significant in the earlier one).

**Figure 5:**
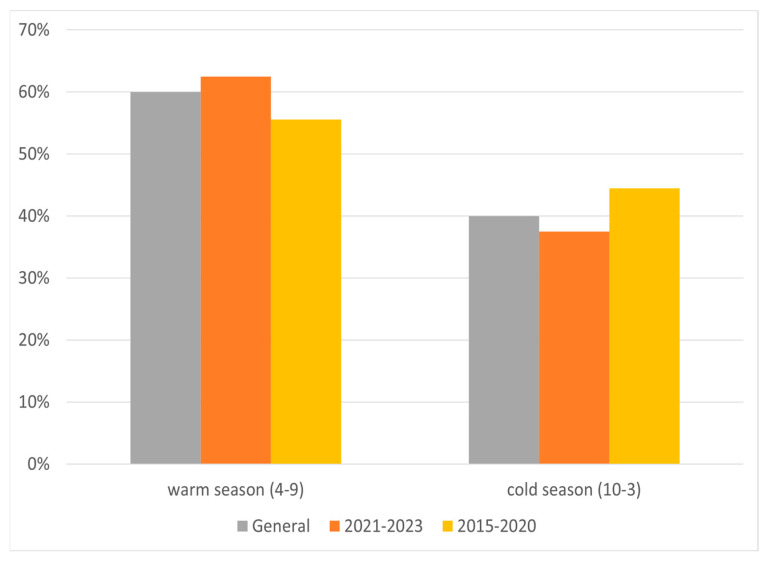
Percentage of relapses according to warm and cold terms in two periods.

Additional analysis of relapse occurrence was made for cold (October to March) and warm (April to September) seasons (Figure 5). In both analyzed periods, about 60% of MS relapses occurred during the warm season.

#### Relationships Between Symptoms of Relapse and Their Seasonal Occurrence

Initial analysis was conducted to search for relationships between symptoms of relapse and the month or season of their occurrence. The results are presented in Table 3. The most significant coincidences (at least 17%) were found for sensory symptoms in January, optic neuritis in March, motor deficits with pyramidal signs in May and June, cerebellar symptoms in March and July, and spinal cord involvement signs in August.

**Figure 6:**
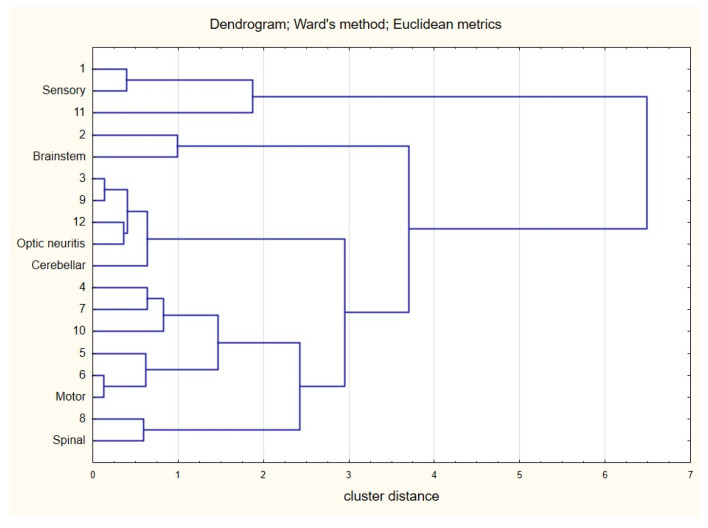
Dendrogram developed using Ward’s method for the variables of month of onset of cast and symptoms (without symptoms of VIII nerve damage).

A correspondence analysis (with Ward’s cluster analysis) of these relationships provided additional findings, with four groups of correlations (Figure 6): one—March, September, and December associated with optic neuritis; two—January—with sensory symptoms; three—April to July and October—with motor deficit with pyramidal signs; and four—August—with spinal cord involvement signs.

### 4.3. Triggering Factors

Potential triggers associated with relapses were analyzed. In the whole study group, such factors were reported by 50 (62.5%) participants, mainly women (Table 4). The most common type of trigger was stressful life event, reported by 25 patients, with a significant predominance at women—21. Respiratory tract infection preceded relapse in 16 cases (including SARS-CoV-2 in 6) and vaccination in 3 cases, reported only by females. Two relapses occurred during pregnancy, two in the postpartum period, and one was preceded by surgery. A total of 13 of the participants of the study reported more than one triggering factor.

On analysis of triggering factors for relapses within two periods (2015–2020 and 2021–2023), we found that stressful life events followed by infections were the most frequent in both periods. Vaccinations prior to relapses were reported only in the years 2021–2023. Significantly more patients denied any triggering factor for relapse in the years 2021–2023 than in 2015–2020 (29 vs. 17 cases).

The time of relapse onset was characterized by a higher mean and maximum daily temperature in comparison with the whole month and in more than 30% these differences corresponded with the first two classes of standardization (i.e., SD > 1). The humidity pattern at the time of relapse onset showed values below the monthly average in more than 70% of cases, in almost 50% corresponding to standards class 5 and 6 (SD < −1). Similarly, in nearly 75% of the times of relapse onset, the probability of rainfall was lower, and in 63%—the degree of insolation was greater than the monthly average (in 26% classified as class 1 and 2 of standardization (SD > 1). Time of relapse onset was also characterized by greater than average fluctuations in atmospheric pressure, in 43% classified as class 1 and 2 (SD > 1) or class 5 and 6 (SD < −1) (Table 5).

## 5. Discussion

Considering a causative role of genetic predisposition and environmental factors (with interactions between them) in the development of MS, environmental/lifestyle issues potentially triggering relapses seem to deserve attention as objects of investigation [4,5,6]. Findings in this field would provide a scope for non-pharmacological interventions which may support the effect of DMT and positively affect general well-being in MS patients [17]. Therefore, in our study we focused on the characteristics of MS relapses and their circumstances, in search of patterns which would improve our understanding of their background and potential targets for therapeutic approaches.

The clinical presentation of MS relapses reported in our study group was heterogenous. Some interesting relationships were observed between relapse symptoms and age or sex of the patients.

Overall, fewer than 10% of participants were under the age of 30. Currently, there is a trend of increasing age in the MS population, including the patients with late disease onset (more than 50 years of age) and those continuing follow-up and treatment. Furthermore, it can be assumed that improved access to disease-modifying therapies (DMTs) for patients newly diagnosed with MS (still mainly young adults) resulted in a reduced number of relapses in this group. Our study demonstrated that relapse-related motor symptoms were more frequently reported in older patients, while optic neuritis and sensory symptoms predominated in the younger ones. Such distinct localization of demyelinating lesions in particular age groups seems unlikely. These differences may result from better compensatory CNS capacity in younger patients, which would prevent clinical manifestation of some new lesions, as well as from the cumulative effect of previously experienced disease-related CNS damage at a later age.

Gender differences in MS clinical pictures are commonly observed. A predominance of women in the MS population (and consequently in our study group) should be taken into account, as well as male sex being considered as adverse prognostic factor for MS course (i.e., because of more disabling symptoms of neurological deficits). Another explanation for our findings (sensory symptoms reported only in women) may be associated with gender differences in the perception of symptoms and attitude towards their management, with women supposedly more focused on even subtle changes in their condition and more prone to seek a neurologist‘s consultation [18].

The findings from studies on the relationships between exposure to stress and clinical activity of MS (including relapse occurrence) are partly contradictory. This is probably due to methodological issues (lack of objective measures for stress perception and heterogeneity of biological markers used) and individual differences in experiencing and coping with stress.

Similarly to some previous studies [19,20,21,22,23,24], we found stressful events as the most frequent potential trigger for relapse identified by the patients, particularly female ones. Gender-related differences in susceptibility to stress have been already described not only in healthy women but also in those suffering from MS [19]. Apart from gender, the type and severity of the stressful event might contribute to their effect upon relapse occurrence [19,20,21,22,23]. Because of the retrospective mode of the study, we were not able to evaluate these aspects more precisely. Our findings indicate the relevant impact of stress on MS activity and thus a need for preventive measures, including psychological and social support for better coping strategies and stress management in MS patients.

Many studies have considered infections as potential triggers for relapses in MS. According to the literature, up to 27% of relapses are preceded by infection, especially a viral one [19,25,26,27,28,29]. However, these relationships remain controversial; some authors claim that infections do not increase the relapse risk [30], while others provide contradictory evidence [31].

In our study, a significant increase in relapses (including those preceded by infections) was reported in the years: 2021–2023. Among these infections (mainly of respiratory tract, with moderate to severe intensity), COVID-19 was diagnosed in six cases; none of the patients required hospitalization due to infection or oxygen therapy. Many studies (also based on the Polish population) from the pandemic period showed that the incidence and severity of COVID-19 (including hospitalization and mortality rate) in MS patients treated with DMTs were similar to the indices for the general population [32,33,34]. Additional factors worth considering in the interpretation of our findings are associated with preventive measures and lockdown restrictions (even more rigorous for MS patients due to their immune status). In the other hand, fear of severe relapse and high-efficacy treatment agents associated with a greater risk of serious infections, as well as stress related to the extraordinary pandemic situation encouraged MS patients to seek hospitalization. What is worth emphasizing is that, in this period, we observed an increased number of patients reporting to health services due to MS and a rise in the amount relapses treated in hospital (before, many patients were treated in the ambulatory system), these factors resulted in greater opportunities to collect all data and ensure its reliability of that time.

Traditionally, vaccinations had also been regarded as a potential trigger for MS relapses [35,36,37,38,39,40,41]. However, pandemic cohort studies on anti-SARS-CoV-2 vaccine efficacy and safety in MS patients treated with DMTs, and subsequent investigation on other vaccinations in view of high efficacy DMTs, provided evidence that the benefits from preventive vaccinations significantly exceed the potential risk of relapse [42,43]. In our study, vaccinations (all against SARS-CoV-2) were identified as a potential trigger for relapse only in three female patients. This is most likely due to the availability and universality of vaccinations against COVID-19 and wider knowledge of patients about risk factors than before.

The climate of the region inhabited by patients with MS, can be defined as “warm–temperate” in accordance with the classic classification by Köppen. Legnica is one of the warmest cities in Poland, where the average annual air temperature in the period 1991–2020 was 9.6 °C. However, this value is not conclusive in a longer timeframe as in the former decades it steadily increased, indicating progressive warming of the climate. For instance, in the consecutive 30-year periods (1951–1980, 1961–1990, 1971–2000, 1981–2010, and 1991–2020) the average temperature consistently increased, being, respectively, 8.1 °C; 8.2 °C; 8.6 °C; 9.0 °C; and 9.6 °C. One of the features of the thermal regime is variability of the average day-to-day air temperature. In 11% of cases, the absolute magnitude of changes in the mean daily temperature exceeds 4 °C, but in extreme situations, the temperature drops from day to day by even 20 °C and increases as much as 15 °C. The average precipitation measured in Legnica between 1990 and 2020 was 630 mm. February displays the lowest monthly total, which is merely 5.4% of the average annual precipitation. In contrast, precipitation in July reaches 14% of the annual total, with the modal value of 89 mm. A characteristic feature of the currently observed warming is that the biggest changes concern the average winter temperatures, while less pronounced and less regular changes are observed in summer. Although quasi-cyclical fluctuations are still observable, i.e., abnormally warm summers alternate with cool ones, the latter are less and less frequent.

Seasonal patterns of MS relapse occurrence have been already explored in some studies, with somehow conflicting results [33,44,45,46,47,48,49,50,51]. These inconsistencies were partly due to various study protocols or tools applied for analysis, but they seem mainly affected by differences in climatic zones depending on the geographical latitude (with greater and more reliable evidence available for the northern hemisphere) [50,51,52]. In meta-analysis of studies conducted in Europe and North America, the peak of relapse incidence was demonstrated in spring and early summer, and a decrease was seen in late summer [51,52]. On the contrary, an increase in relapse activity was observed during winter months (December–January) in Saudi Arabia and Brazil [50,51,52,53]. This pattern is also supported by MRI data. In the study from the Center for Neurological Imaging of Boston, the frequency and extent of active (gadolinium contrast-enhanced in T1) lesions and new lesions in T2 showed seasonal fluctuations and were significantly higher during the spring and summer season (March–August) than during the rest of the year. These neuroimaging data correlated significantly with clinical activity [54]. The observed pattern is suggestive of a modulating role of seasonally changing environmental factors.

Moreover, there is some evidence for seasonal changes in metabolic and immunologic activity, putatively linked with the disease course. Higher serum concentration of overlapping metabolites was observed in MS patients during spring and fall [55] and a significant increase in pro-inflammatory cytokine production (such as Il-6, TNF-alfa, and IFN-gamma) was seen from spring to summer. Particularly, the latter findings seem relevant, considering principal role of these cytokines as mediators of MS relapse [55]. On the other hand, seasonal fluctuations of endogenous vitamin D levels may contribute to anti-inflammatory effects (inhibiting pro-inflammatory cytokines and promoting regulatory T-cells).

The findings from our study are similar to the typical observations for the northern hemisphere. In general, almost a twice as high relapse occurrence was reported in warm seasons, especially in May and June. On separate analysis of the two periods (2015-20 and 2021-23), no relapses in winter were noted during the first one.

The above pattern of relapse occurrence may seem surprising, considering the relevant role of vitamin D3 deficiency and limited exposure to sunlight in the development and activity of MS. Supplementation of vitamin D, especially in autumn and winter months is recommended to support effects of DMTs upon the disease course, including the prevention of relapses [22,50,51,52]. However, the impact of these environmental factors on MS seems rather a long-term one, while exposure to heat may more directly affect the occurrence of relapses. High ambient temperature contributes to conduction block in demyelinated axons and consequently new or recurrent symptoms of neurological deficit, which is known as the Uhthoff phenomenon [13,14,49,56,57,58,59,60]. Heat-induced fatigue is also often observed in MS patients. It is worth highlighting that the relapses included in our study analysis were verified by a specialist (symptoms of new or increased neurological deficit lasting for at least 24 h) and thus distinguished from temporary worsening during exposure to heat (which resolves after cooling). Another argument in favor of the fact that temperatures have an impact on the course of disease is the utility of cryotherapy in adjuvant care in MS. It has an influence on reducing fatigue, diminishing spasticity, and has undisputed analgesic effects. In addition, using cryotherapy revealed significant improvements in depressive syndrome, quality of life, and disease acceptance [61,62,63].

On the other hand, occasional increases in relapse occurrence in autumn and spring months might be attributed to seasonal frequency of infections. As discussed above, these relationships were complex, particularly during the SARS-CoV-2 pandemic. Subsequent waves of increased COVID-19 incidence thoroughly modified the typical annual pattern of infections. Lockdown and preventive restrictions (supposedly followed by MS patients more rigorously and for longer periods) might have reduced exposure to infections and weather conditions but were undoubtedly associated with increased burden of stress. Furthermore, fear of hospitalization (increased risk of infection in healthcare settings) possibly resulted in a lower rate of reported relapses. Due to these complex links, our findings should be interpreted cautiously.

Seasonal patterns of relapse occurrence in our study indicated the potential role of high outdoor temperatures in triggering relapses. Current climate data and the consensus among climate scientists indicate that temperatures year-round, particularly during the hottest months, have been increasing over the past decade. Analysis of MS relapse occurrence in view of these climate trends certainly deserves attention. Berntsson et al. reviewed 24 studies on the effects of environmental heat on seasonal variations in MS course; in more than 60%, the authors concluded that heat worsens clinical symptoms and the rate of hospitalizations. However, large prospective studies seem necessary to outline these relationships reliably. A range of factors contributing to relapse occurrence (as described above) and the retrospective nature of our study do not allow us to draw conclusions about the impact of global warming upon MS course [64,65].

In additional analysis, we undertook more precise evaluations not only of temperature measurements, but also of other meteorological parameters potentially associated with relapses. Few available studies on this topic provide inconsistent results, either suggesting a link between relapse occurrence and increased temperature, insolation, and low humidity [58,59] or denying such relationships [58,59,61]. After a detailed analysis, we identified a specific profile of meteorological conditions directly preceding reported relapses. Its basic features included high average and maximum daily temperature, greater insolation, low relative humidity with lack of rainfall, as well as daily fluctuations in atmospheric pressure. The reliability of these findings is supported by objective measurements provided by local meteorological stations.

The analysis of relapsing-remitting MS included events that occurred between 2015 and 2023, but approximately 75% of them concerned the period between 2020 and 2023. These were indeed very warm years, with an average annual temperature 1.0–1.5 degrees Celsius higher than the 30-year norm. However, if we focus our attention on individual months, the picture is no longer so clear-cut. When analyzing the annual course of relapsing-remitting MS, the clear predominance of April, May, and June emerges. These three months (in 2020–2023) were characterized by very diverse thermal conditions. April and May were usually significantly cooler than the long-term average (by as much as 1.5–2.0 degrees Celsius), while June was significantly warmer (by 1.5 to 2.5 degrees Celsius). Since we do not observe a clear shift in relapsing-remitting MS from a cool spring to a warm summer, the conclusion is that there is no strict and direct link between these events and the upward trend in air temperature.

The strength of our study is associated with the multidimensional characteristics of relapses and their circumstances in MS patients, considering their interactions and individual differences. We believe that seasonal patterns of relapse occurrence and potentially associated weather conditions, which have been rarely analyzed so far, deserve attention in this context. The limitations of the study include the relatively small sample size and retrospective mode with potentially incomplete data in medical records. Omitting findings from MRI and information about DMT efficacy (due to their limited availability) might have also affected the relevance of the final results. Potential triggering factors for relapses might have been underestimated because of a recall bias and lack of quantitative assessment of their severity (e.g., regarding infection or stressful events). Also, distinguishing between a relapse and Uhthoff phenomenon, despite careful differentiating, was challenging and may have turned out to be underestimated. Further prospective observations in large groups of MS patients seem necessary, including precise determination of relapse features and potential triggers in order to establish their relevant relationships with future clinical implications.

## 6. Conclusions

In conclusion, retrospective analysis of relapse characteristics in a group of MS patients showed that the clinical manifestation of relapses depended on age and gender. Stressful life events and infections were the most frequent potential triggers for relapses. Observation of seasonal occurrence of relapses and associated weather conditions revealed summer/spring months, in particular periods with high temperature, low humidity, and variable atmospheric pressure as potential contributors. Relapse occurrence is supposed to be affected by interactions of environmental and individual patient-related factors. Better recognition of these issues within future investigations could be considered in the complex approach to the management of MS outcomes.

## Figures and Tables

**Table 1:** Symptoms of a relapse according to gender.

Symptoms	Females		Males		*p*-Value
Sensory	7	12%	0	0%	0.229
Cerebellar	8	14%	2	10%	0.924
Optic neuritis	12	20%	2	10%	0.432
N. VIII palsy	1	2%	0	0%	0.587
Motor	26	44%	12	57%	0.303
Brainstem	1	2%	1	5%	0.968
Spinal	4	7%	4	19%	0.108
SUMMARY	59	100%	21	100%	

**Table 2:** Symptoms of a relapse according to age and gender.

Symptoms	<30 Years			30–39 Years			40–49 Years			>50 Years		
	F	M	SUM	F	M	SUM	F	M	SUM	F	M	SUM
Sensory	3%	0%	3%	3%	0%	3%	3%	0%	3%	2%	0%	1%
Cerebellar	0%	0%	0%	3%	0%	3%	3%	5%	4%	7%	5%	6%
Optic neuritis	3%	0%	3%	12%	5%	10%	2%	5%	3%	3%	0%	3%
N.VIII palsy	2%	0%	1%	0%	0%	0%	0%	0%	0%	0%	0%	0%
Motor	2%	0%	1%	10%	24%	14%	17%	10%	15%	15%	24%	18%
Brainstem	2%	0%	1%	0%	0%	0%	0%	0%	0%	0%	5%	1%
Spinal	0%	0%	0%	0%	10%	3%	2%	5%	3%	5%	5%	5%
SUM	12%	0%	9%	29%	38%	31%	27%	24%	26%	32%	38%	34%

The abbreviations: F—females, M—males, SUM—summary.

**Table 3:** Symptoms of a relapse according to month.

	Month	Brainstem	Sensory	Optic Neuritis	Motor Deficits	Cerebellar	Spinal	n.VIII Palsy	Summary
Amount	January	0	3	1	1	0	0	0	5
Percentage		0%	43%	7%	3%	0%	0%	0%	
Amount	February	1	0	1	3	0	1	0	6
Percentage		50%	0%	7%	8%	0%	13%	0%	
Amount	March	0	0	3	3	2	0	1	9
Percentage		0%	0%	21%	8%	20%	0%	100%	
Amount	April	0	1	2	5	1	0	0	9
Percentage		0%	14%	14%	13%	10%	0%	0%	
Amount	May	1	0	0	7	1	1	0	10
Percentage		50%	0%	0%	18%	10%	13%	0%	
Amount	June	0	0	2	9	0	1	0	12
Percentage		0%	0%	14%	24%	0%	13%	0%	
Amount	July	0	1	0	4	2	1	0	8
Percentage		0%	14%	0%	11%	20%	13%	0%	
Amount	August	0	0	1	1	1	2	0	5
Percentage		0%	0%	7%	3%	10%	25%	0%	
Amount	September	0	0	1	1	1	0	0	3
Percentage		0%	0%	7%	3%	10%	0%	0%	
Amount	October	0	1	1	1	1	1	0	5
Percentage		0%	14%	7%	3%	10%	13%	0%	
Amount	November	0	1	0	0	0	1	0	2
Percentage		0%	14%	0%	0%	0%	13%	0%	
Amount	December	0	0	2	3	1	0	0	6
Percentage		0%	0%	14%	8%	10%	0%	0%	

**Table 4:** Triggering factors according to gender.

Risk Factor	<30 Years		30–39 Years		40–49 Years		>50 Years	
	F	M	F	M	F	M	F	M
Stress	10%	0%	16%	6%	19%	19%	23%	0%
Infection	6%	0%	6%	6%	10%	0%	6%	0%
COVID 19	0%	0%	10%	0%	3%	0%	6%	0%
Vaccination	0%	0%	0%	0%	3%	0%	6%	0%
Other	0%	0%	10%	0%	0%	6%	6%	0%
No risk factor	13%	0%	29%	38%	26%	19%	32%	44%

**Table 5:** Characteristics of meteorological parameters for times of relapse onset.

Meteorological Parameter	Time of Relapse Onset (% of Cases in Relation to the Monthly Average) (±Above or Below Average, Respectively)
Maximum daily temperature	+63%
Average daily temperature	+66%
Relative humidity	−71%
Wind speed	Close to the average
Atmospheric pressure	Close to the average
Daily precipitation total	−74%
Insolation	+63%
Daily atmospheric pressure amplitude	A similar share of extreme standardization classes 1 and 2 and 5 and 6–43% in total

## Data Availability

The data presented in this study are available on request from the corresponding author.
